# Identification of circRNA–miRNA–mRNA networks contributes to explore underlying pathogenesis and therapy strategy of gastric cancer

**DOI:** 10.1186/s12967-021-02903-5

**Published:** 2021-05-28

**Authors:** Zhijie Dong, Zhaoyu Liu, Min Liang, Jinhui Pan, Mingzhen Lin, Hai Lin, Yuanwei Luo, Xinke Zhou, Wenxia Yao

**Affiliations:** Guangzhou Key Laboratory of Enhanced Recovery after Abdominal Surgery, The Fifth Affiliated Hospital of Guangzhou Medical University, Guangzhou Medical University, Guangzhou, China

**Keywords:** Gastric cancer, circRNA, ceRNA, Cmap

## Abstract

**Background:**

Circular RNAs (circRNAs) are a new class of noncoding RNAs that have gained increased attention in human tumor research. However, the identification and function of circRNAs are largely unknown in the context of gastric cancer (GC). This study aims to identify novel circRNAs and determine their action networks in GC.

**Methods:**

A comprehensive strategy of data mining, reverse transcription-quantitative polymerase chain reaction (RT-qPCR) and computational biology were conducted to discover novel circRNAs and to explore their potential mechanisms in GC. Promising therapeutic drugs for GC were determined by connectivity map (CMap) analysis.

**Results:**

Six overlapped differentially expressed circRNAs (DECs) were screened from selected microarray and RNA-Seq datasets of GC, and the six DECs were then validated by sanger sequencing and RNase R treatment. Subsequent RT-qPCR analysis of GC samples confirmed decreased expressions of the six DECs (hsa_circ_0000390, hsa_circ_0000615, hsa_circ_0001438, hsa_circ_0002190, hsa_circ_0002449 and hsa_circ_0003120), all of which accumulated preferentially in the cytoplasm. MiRNA binding sites and AGO2 occupation of the six circRNAs were predicted using online databases, and circRNA–miRNA interactions including the six circRNAs and 33 miRNAs were determined. Then, 5320 target genes of the above 33 miRNAs and 1492 differently expressed genes (DEGs) from The Cancer Genome Atlas (TCGA) database were identified. After intersecting the miRNA target genes and the 889 downregulated DEGs, 320 overlapped target genes were acquired. The Kyoto Encyclopedia of Genes and Genomes enrichment analysis indicated that these target genes were related to two critical tumor-associated signaling pathways. A protein–protein interaction network with the 320 target genes was constructed using STRING, and fifteen hubgenes (ATF3, BTG2, DUSP1, EGR1, FGF2, FOSB, GNAO1, GNAI1, GNAZ, GNG7, ITPR1, ITPKB, JUND, NR4A3, PRKCB) in the network were identified. Finally, bioactive chemicals (including vorinostat, trichostatin A and astemizole) based on the fifteen hubgenes were identifed as therapeutic agents for GC through the CMap analysis.

**Conclusions:**

This study provides a novel insight for further exploration of the pathogenesis and therapy of GC from the circRNA-miRNA-mRNA network perspective.

**Supplementary Information:**

The online version contains supplementary material available at 10.1186/s12967-021-02903-5.

## Background

Circular RNAs (circRNAs) are a newly identified class of noncoding RNAs (ncRNAs) that have recently elicited increased attention [1, 2]. CircRNAs are formed by back-splicing of a downstream splice donor site to an upstream splice acceptor site, thus producing a covalently closed RNA molecule. Lacking 5′ caps and 3′ poly (A) tails, circRNAs have not received significant attention for a long time; recently, with the application of high-throughput sequencing, a growing number of circRNAs have been unveiled [3]. Studies on circRNAs have revealed that they are structurally stable, presumably because their lack of free ends is resistant to exonuclease activity, which enables circRNAs to serve as a new class of diagnostic or prognostic biomarkers of diseases. Furthermore, emerging lines of studies have revealed that some circRNAs play important roles in physiological and pathological conditions including malignant tumors, and they may provide new potential therapeutic targets [4].

Gastric cancer (GC) is the fifth most common cancer and the fourth-leading cause of cancer-related deaths worldwide [5] and is thus a global cancer burden. Although diagnosis and treatment have improved over the last decades, the prognosis remains poor and the 5-year survival rate remains low in patients with GC [6]. Therefore, the discovery of effective biomarkers and therapeutic targets is of great importance. Competing endogenous RNA (ceRNA) refers to RNAs that sequester or sponge miRNAs to regulate mRNA transcripts containing common miRNA recognition elements (MREs) [7]. Recent studies reported that a large amount of conserved MREs present in circRNAs [8], making circRNAs a new research hotspot in the field of ceRNA. CircRNAs have been found to participate in various biological processes in GC by working as ceRNAs [9–11]. However, the identification and function of circRNAs in GC still require further investigation.

In the current study, data mining and bioinformatics analysis were combined to identify novel circRNAs and to investigate the underlying mechanism of circRNA-miRNA-mRNA network in GC (Additional file 2: Figure S1). First, we collected GC-related circRNA microarray and RNA-Seq datasets from the Gene Expression Omnibus (GEO) databases and PubMed publications and screened overlapped differentially expressed circRNAs (DECs). Then we validated these overlapped DECs by sanger sequencing and RNase R treatment, and verified their expression via reverse transcription-quantitative polymerase chain reaction (RT-qPCR) using GC samples. Further subcellular localization analysis and AGO2-binding sites mining indicated that these DECs function as ceRNAs. After predicting the sponge miRNAs of the DECs and miRNA target genes, we constructed circRNA-miRNA-mRNA networks; Kyoto Encyclopedia of Genes and Genomes (KEGG) enrichment analysis on the target genes were conducted to investigate the potential pathogenesis of GC. Then, a protein–protein interaction (PPI) network with target genes was set up and hubgenes were determined. Moreover, we performed a connectivity map (CMap) analysis based on the hubgenes to identify potential bioactive compounds, which provides possible alternatives and chemotherapeutics for the treatment of GC patients.

## Methods

### Screening of DECs in GC from GEO database and PubMed publications

The microarray and RNA-Seq data was obtained from GEO databases (http://www.ncbi.nlm.nih.gov/gds/) [12] and PubMed publications. Three published microarray datasets that analyzed circRNA expression profile between GC tissues and matched nontumor tissues were retrieved from GEO database until June 2019 (Table 1), and the microarray dataset reported by Dang et al. was selected for further analysis since it has the largest number of identified circRNAs among the three datasets [13]. And the RNA-Seq dataset reported by Chen et al. [11] was selected for the same reason that it has the largest number of identified circRNAs among RNA-Seq datasets until June 2019. Limma [14], a Bioconductor package, was used to screen DECs, and “|Log2 (fold change) |> 1 and *P*-value < 0.05” were defined as the criteria for the DECs screening. The overlapped DECs were identified by intersecting the DECs from the aforementioned two datasets.

**Table 1:** Basic information of microarray and RNA-Seq datasets

Data source	Platform	Authors	Publication	Year	Sample size (T/N)	circRNA Number
Microarray datasets from GEO						
GSE100170	GPL23259	Dang et al	Sci Rep	2017	5/5	62,998
GSE89143	GPL19978	Shao et al	Cancer Med	2017	3/3	5,396
GSE78092	GPL21485	Huang et al	Mol Med Rep	2017	3/3	13,617
RNA-Seq datasets from PubMed publications						
PMID: 27,986,464	GPL11154	Chen et al	Cancer Lett	2017	3/3	15,623

*GEO* Gene Expression Omnibus, *T* tumor, *N* normal

### Cell culture and tissue specimens

GES-1-T cells were constructed in the previous study [15], and were cultured in Dulbecco′s modified Eagle′s medium (DMEM) with 10% fetal bovine serum (FBS) and 1% penicillin–streptomycin. Human GC cell lines HGC-27 and MGC-803 were cultured in RPMI 1640 medium with 10% FBS and 1% penicillin–streptomycin. All cells were incubated in a humidified atmosphere containing 5% CO2 at 37 °C. Eight pairs of fresh frozen GC tissues and corresponding adjacent normal tissues were obtained from patients who underwent surgery at the Fifth Affiliated Hospital of Guangzhou Medical University. All samples used in this study were collected with patients’ consent. The present study was approved by the Ethics Committee of the Fifth Affiliated Hospital of Guangzhou Medical University.

### RNA isolation, nuclear-cytoplasmic fractionation and RNase R treatment

Total RNA was extracted from cells and tissues using TRIzol (Invitrogen) according to the manufacturer’s instructions. Nuclear and cytoplasmic RNAs were isolated with PARIS Kit (Invitrogen, AM1921) following the manufacturer’s instruction. For RNase R treatment, total 10 ug RNA was incubated for 20 min at 37 °C with or without 2 units of RNase R (Epicentre, RNR07250); the resulting RNA was then purified with an RNeasy MinElute Cleanup Kit (Qiagen, 74,204).

### RT-qPCR

RNA was reversely transcribed into cDNA using PrimeScript™ RT reagent Kit with gDNA Eraser (TaKaRa, RR047A) in the presence of random hexamers (TaKaRa). qPCR was conducted using a TB Green® Premix Ex Taq™ II Kit (TaKaRa, RR820A) on the Applied Biosystems 7500 Real Time PCR System. β-actin served as an internal control. The specific primers of circRNAs were listed in Table 2.

**Table 2:** Primer sequences for reverse transcription-quantitative polymerase chain reaction

Gene ID	Primer sequence		Product length
	Forward (5**′**-3**′**)	Reverse (5**′**-3**′**)	
hsa_circ_0000390	GCACAAGAACGCTACCTTTTCTTA	TTTTTTAGCATCATTCCAGTCCAG	165
hsa_circ_0000615	GAAAGTCAAGTCTGAAAAGCAATG	ACATCTTAGAGTCAACGTCCCAC	150
hsa_circ_0001438	GTAATCAACGTAAGAGAGAATTCGG	TGGCTGAGGACGCTCTGAA	157
hsa_circ_0002190	TCAATGGCTCCCTTTATGTCTT	CCATGCAGCACAACTCTCTTCT	149
hsa_circ_0002449	CCTTATCCACCACAACCAATG	ATCGGGGACCTGTAAAGTGC	152
hsa_circ_0003012	CATCCAGATTATCCACTGCAATC	TTCGGGAACCACAGCAGAT	146

### Prediction of MREs

MiRNA binding sites in DECs were predicted using web tools CircInteractome (https://circinteractome.nia.nih.gov/) [16] and Circbank (www.circbank.cn) [17] respectively. Only MREs predicted consistently by both Circbank and CircInteractome were used for further analysis.

### AGO2-binding sites from cross-linking immunoprecipitation (CLIP) data sets

The evidence for AGO2-binding sites was acquired from published online CLIP data sets on doRiNA database (http://dorina.mdc-berlin.de) [18]. These data sets contain AGO2 PAR-CLIP or HITS-CLIP data from several cell lines. After downloading the available data sets, AGO2-binding sites of circRNA genomic region were acquired.

### Prediction of miRNA target genes

MiRNAs targeted genes were predicted using miRWalk 2.0 [19], which involves 12 algorithms (miRWalk, Microt4, miRanda, mirbridge, miRDB, miRMap, miRNAMap, Pictar2, PITA, RNA22, RNAhybrid and Targetscan). Target genes identified by at least eight algorithms were chosen.

### Collection of differently expressed genes (DEGs) of GC from The Cancer Genome Atlas (TCGA)

RNA-Seq data of 415 gastric adenocarcinoma samples and 35 normal tissues was downloaded from the TCGA database. DEGs were determined by DEseq [20], a Bioconductor package; with the cutoff of gene reads > 1, total 12,408 genes were inclusive in all samples, and 1492 genes were differentially expressed with the criteria of |log2 (fold change) |> 1 and *P*-value < 0.05. Among the 1492 genes, 603 genes were upregulated and 889 genes were downregulated.

### Construction of circRNA–miRNA–mRNA networks

The predicted miRNA target genes and the downregulated DEGs from TCGA were intersected to obtain the overlapped genes. Then, circRNA–miRNA–mRNA networks were constructed and visualized using the Cytoscape3.6.1 software [21].

### Establishment of a PPI network and identification of hubgenes

The PPI network directly indicate the protein interactions established using STRING (version 11.0) [22]. Seven active interaction sources were selected: textmining, experiment, database, co-expression, neighborhood, gene fusion, and co-occurrence. Confident score was set to medium (score 0.400). The results were visualized by the Cytoscape3.6.1 software. Next, the “Molecular Complex Detection” (MCODE), a plugin of Cytoscape, was employed to find the highly interacted hubgene clusters.

### Bioinformatic analysis tools

The KEGG Pathway based data integration and visualization was carried out using Pathview Web server (https://pathview.uncc.edu/) [23]: expression data of 320 target genes in ENSEMBL ID type of GC tissues (as sample) and normal tissues (as control) were loaded into the Pathview input tool, and the output suffix was set as KEGG pathview. Venn diagrams were generated using an online analysis platform (http://bioinfogp.cnb.csic.es/tools/venny/index.html).

### CMap analysis

CMap (https://portals.broadinstitute.org/CMap/) [24, 25] is a collection of gene expression profiles from cultured human cells treated with bioactive small molecules; CMap helps scientists to discover functional connections among diseases, genetic perturbation, and drug action. Downregulated tags of the fifteen hubgenes were loaded into the CMap online tool, searching against 6100 treatment-controls (instances) in which 1309 bioactive molecules were involved. A connectivity score ranging from − 1 to 1 was used to estimate the closeness between query signatures and compounds: a positive score indicates a promotive effect of compound on the query signatures, whereas a negative score denotes an inverse effect of compound on the query signatures.

### CircRNA overexpression and cell transfection

CircRNA overexpression plasmids were cloned with the pCD2.1-ciR vector, and all the circRNA sequence was confirmed by Sanger sequencing. For transient transfection, GC cells were transfected with the reagents using Lipofectamine™ 3000 (Invitrogen) according to the manufacturer’s instructions.

### Cell counting Kit-8 (CCK-8) assay

Cell proliferation was assayed by CCK-8 (Dojindo, Tokyo) according to the manufacturer’s protocols. GC cells in logarithmic growth were plated in each well of a 96-well plate. Then, on the indicated time, 10 μl of CCK-8 solution was added to each well. Following 1 h of incubation at 37 °C, the absorbance of each well was measured at 450 nM by Synergy 2 microplate reader (BioTek, Winooski, VT, USA).

## Results

### Identification of six downregulated DECs in GC

**Fig. 1:**
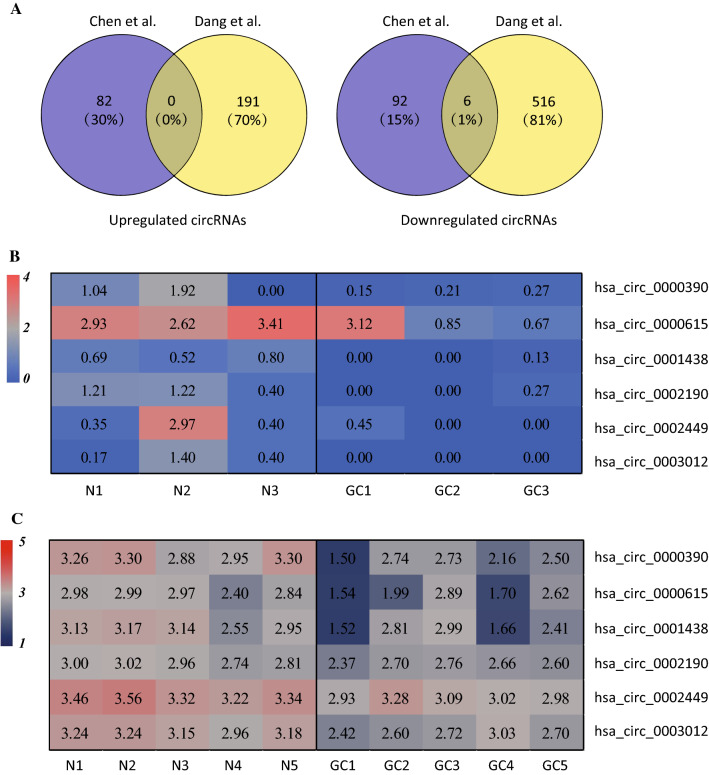
Identification of differentially expressed circRNAs (DECs) in gastric cancer (GC) using microarray and RNA-Seq datasets. **A** Venn diagram showing overlapped circRNAs that were upregulated or downregulated in studies performed by Chen et al. and Dang et al., respectively. **B**, **C** Heatmap for the six DECs in individual microarray or RNA-Seq datasets. Red indicates a higher expression and blue indicates a lower expression

First, we collected GC-related circRNA microarray and RNA-Seq datasets from GEO databases and PubMed publications and screened overlapped DECs. As displayed in Table 1, one microarray dataset from GEO databases (GSE100170) [13] and one RNA-Seq dataset from PubMed publications (PMID: 27986464) [11] were selected for analysis in this study since these two datasets had the largest numbers of identified circRNAs respectively. A total of 180 DECs were identified in the study performed by Chen et al. [11], including 82 upregulated circRNAs and 98 downregulated circRNAs (Fig. 1A); a total of 713 DECs were identified in the study by Dang et al. [13], of which 191 circRNAs were upregulated and 522 circRNAs were downregulated (Fig. 1A).

Further Venn diagram analysis showed that there were six overlapped downregulated circRNAs and none overlapped upregulated circRNAs in both studies (Fig. 1A). The six overlapped downregulated circRNAs were hsa_circ_0000390, hsa_circ_0000615, hsa_circ_0001438, hsa_circ_0002190, hsa_circ_0002449 and hsa_circ_0003120. The expression of the six DECs in individual microarray or RNA-Seq dataset was displayed: in the study by Chen et al. (Fig. 1B), six DECs was significantly lower with good consistency in GC tissues comparing to the matched nontumor tissues; downregulation of the six DECs in GC tissues was also observed in the study by Dang et al. (Fig. 1C).

### Validation of the six DECs and verification of their downregulation with GC samples

**Fig. 2:**
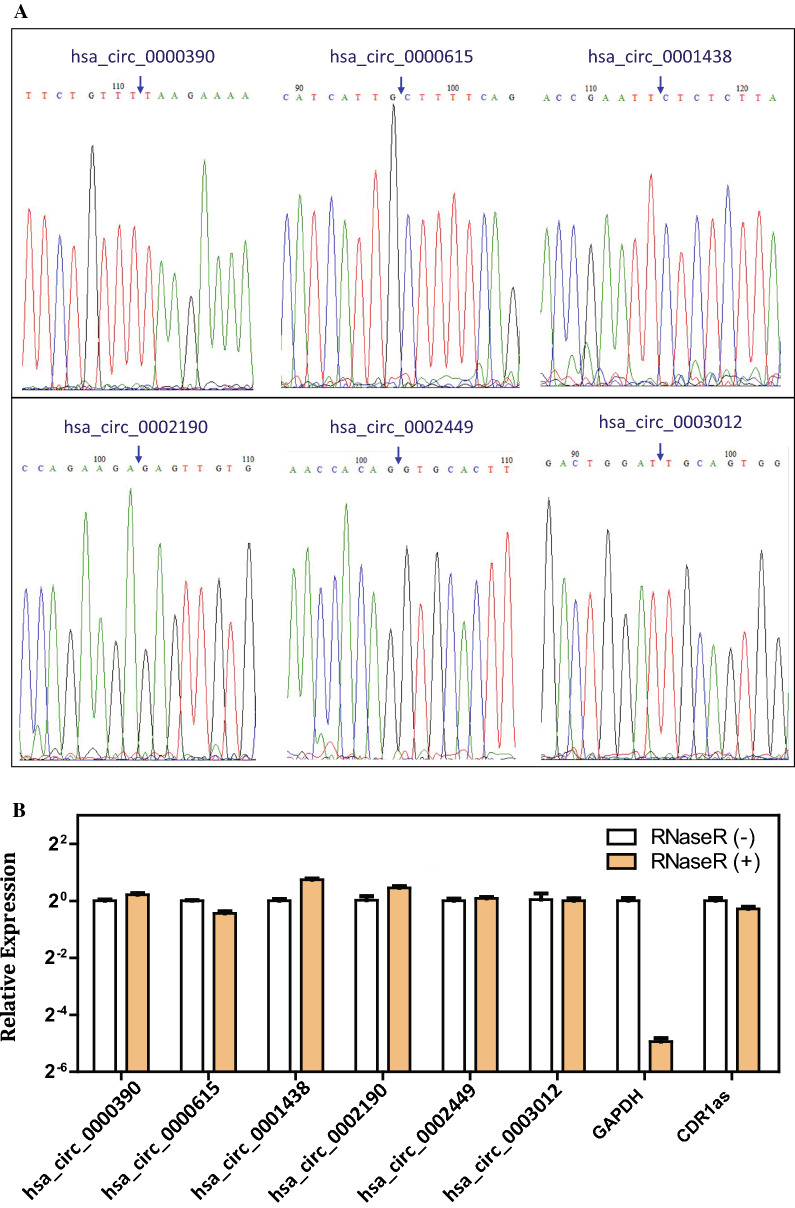
Validation of the six DECs by Sanger sequencing and RNase R treatment. **A** Head-to-tail splicing in the RT-qPCR product of the six DECs by Sanger sequencing. Blue arrow indicates the back-splicing of DECs. **B** qRT-PCR analysis of the abundance of the six DECs in MGC-803 cells after treatment with RNase R. The amounts were normalized to the value measured in the untreated group

We then analyzed the six DECs. The principal characteristics of the six DECs were listed in Table 3. Divergent primers (Table 2) were designed against the six DECs. By sanger sequencing of the RT-PCR product, head-to-tail splicing in the six DECs was confirmed (Fig. 2A). Moreover, after the treatment of RNase R, a processive 3′ to 5′ exonuclease, the six DECs was apparently enriched, which was consistent with the positive control CDR1as [26]; however, the abundance of linear GAPDH mRNA was apparently decreased (Fig. 2B). These results confirmed the circular characteristics of the six DECs.

**Table 3:** Essential characteristics of the six differently expressed circRNAs

CircRNA	Type	Chr	Start	End	Strand	Spliced length	Gene symbol
hsa_circ_0000390	Exonic	chr12	32760890	32,764,217	+	345	FGD4
hsa_circ_0000615	Exonic	chr15	64,791491	64,792,365	+	874	ZNF609
hsa_circ_0001438	Exonic	chr4	128,995614	128999117	+	294	LARP1B
hsa_circ_0002190	Exonic	chr7	129760588	129762042	+	304	KLHDC10
hsa_circ_0002449	Exonic	chr5	139574030	139574237	+	207	C5orf32
hsa_circ_0003012	Exonic	chr12	105303432	105322472	−	830	SLC41A2

**Fig. 3:**
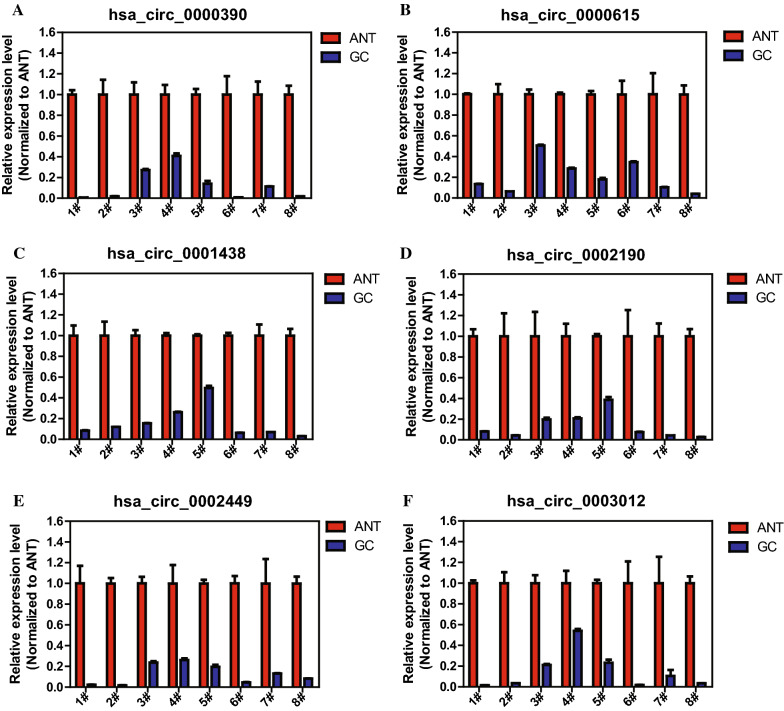
Expression of the six DECs in GC by RT-qPCR analysis: **A** hsa_circ_0000390, **B** hsa_circ_0000615, **C** hsa_circ_0001438, **D** hsa_circ_0002190, **E** hsa_circ_0002449, and **F** hsa_circ_0003012. The amounts of circRNAs were normalized to the value measured in adjacent normal tissues. ANT, adjacent normal tissues; GC, gastric cancer

Then, RT-qPCR analysis was performed with 8 pairs of GC samples and adjacent nontumor tissues to verify the induced expression of the six DECs. As shown in Fig. 3A–F, expression of the six DECs were dramatically downregulated in GC tissues than adjacent normal tissues.

### Identification of circRNA-miRNA interactions and AGO2-binding sites

**Fig. 4:**
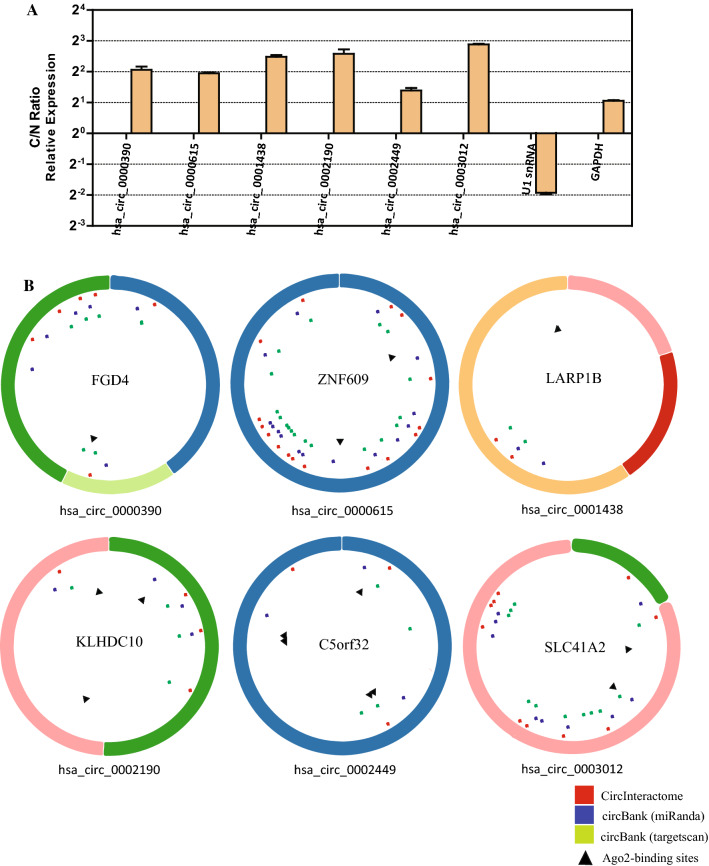
Subcellular distribution of the six DECs and identification of circRNA-miRNA interactions and AGO2 occupation. **A** qRT-PCR analysis of subcellular distribution of the six DECs. The amounts of circRNAs were normalized to the value measured in the nucleus. C/N ratio, the ratio of cytoplasm to nucleus. **B** Structural patterns of the six DECs with predicted MREs and AGO2-binding sites. Red dots represent MREs predicted by CircInteractome while blue dots and green dots represent MREs predicted by Circbank using miRanda and Targetscan, respectively. Black triangles represent AGO2-binding sites

We subsequently determined the potential function of the six DECs in GC. Since the function of circRNAs was associated with their subcellular localization, RT-qPCR analysis of nuclear or cytoplasmic fractions of GC cell lines was performed to evaluate the subcellular distribution of the six DECs. As expected, U1 snRNA was enriched in the nuclear while the coding GAPDH mRNA was primarily localized in cytoplasm; as demonstrated, all the six DECs were found predominantly in the cytoplasm (Fig. 4A).

Given that increasing circRNAs have been reported to play important roles in tumor through absorbing miRNA and that the six DECs accumulated preferentially in the cytoplasm, three online databases were used to determine whether the six DECs function as ceRNAs in GC. Herein CircInteractome and Circbank were utilized to predict potential target miRNAs of the six DECs, and only circRNA–miRNA interactions identified by both Circbank and CircInteractome were used (Fig. 4B), which are also shown in Additional file 2: Table S1. A total of 36 circRNA-miRNA interactions consisting of 6 circRNAs and 33 miRNAs (3 overlapped miRNAs) were identified. Moreover, online AGO2 PAR-CLIP or HITS-CLIP data was downloaded from doRiNA database [18] to ascertain whether AGO2 occupies in the region of the six DECs. The result demonstrated that all the six DECs contain AGO2 binding sites and 4 out of 6 circRNAs contain at least two AGO2 binding sites (Fig. 4B).

Taken together, the six DECs possibly function by sponging miRNAs.

### Construction of circRNA-miRNA-mRNA networks and KEGG pathway analysis of the target genes

**Fig. 5:**
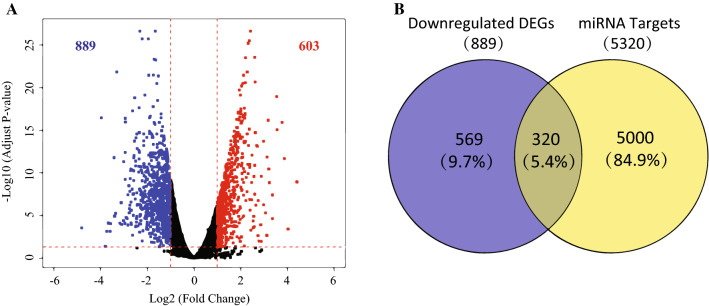
Identification of 320 overlapped genes that probably play critical roles in GC. **A** Volcano plot of the differentially expressed genes (DEGs) in GC based on the data from The Cancer Genome Atlas (TCGA). Significantly upregulated circRNAs are indicated in red and downregulated circRNAs are indicated in blue (FC ≥ 2.0 or FC ≤ 0.5 and p < 0.05). **B** Venn diagram for the intersection between downregulated DEGs and miRNA target genes

To further illustrate the potential ceRNA mechanisms of the six DECs in GC, prediction of miRNA target genes was performed by miRWalk with the criteria of that target genes identified by at least 8 out of 12 algorithms were chosen for further analysis, and a total of 5320 target genes of the above-mentioned 33 miRNAs were obtained. In addition, 1492 DEGs in GC were obtained from TCGA (Additional file 1), of which 603 DEGs were upregulated and 889 DEGs were downregulated (Fig. 5A). After intersecting the 5320 miRNA target genes and the 889 downregulated DEGs from TCGA, 320 overlapped target genes were acquired (Fig. 5B), which probably serve crucial roles in GC.

Then, circRNA-miRNA-mRNA networks were generated using a combination of data from circRNA-miRNA interactions and the overlapped 320 target genes, which indicated an overall perspective of regulation networks consisting of six circRNAs, 33 miRNAs and 320 target mRNAs. The ceRNA networks with six DECs were shown in Additional file 2: Figure S2–3.

**Fig. 6:**
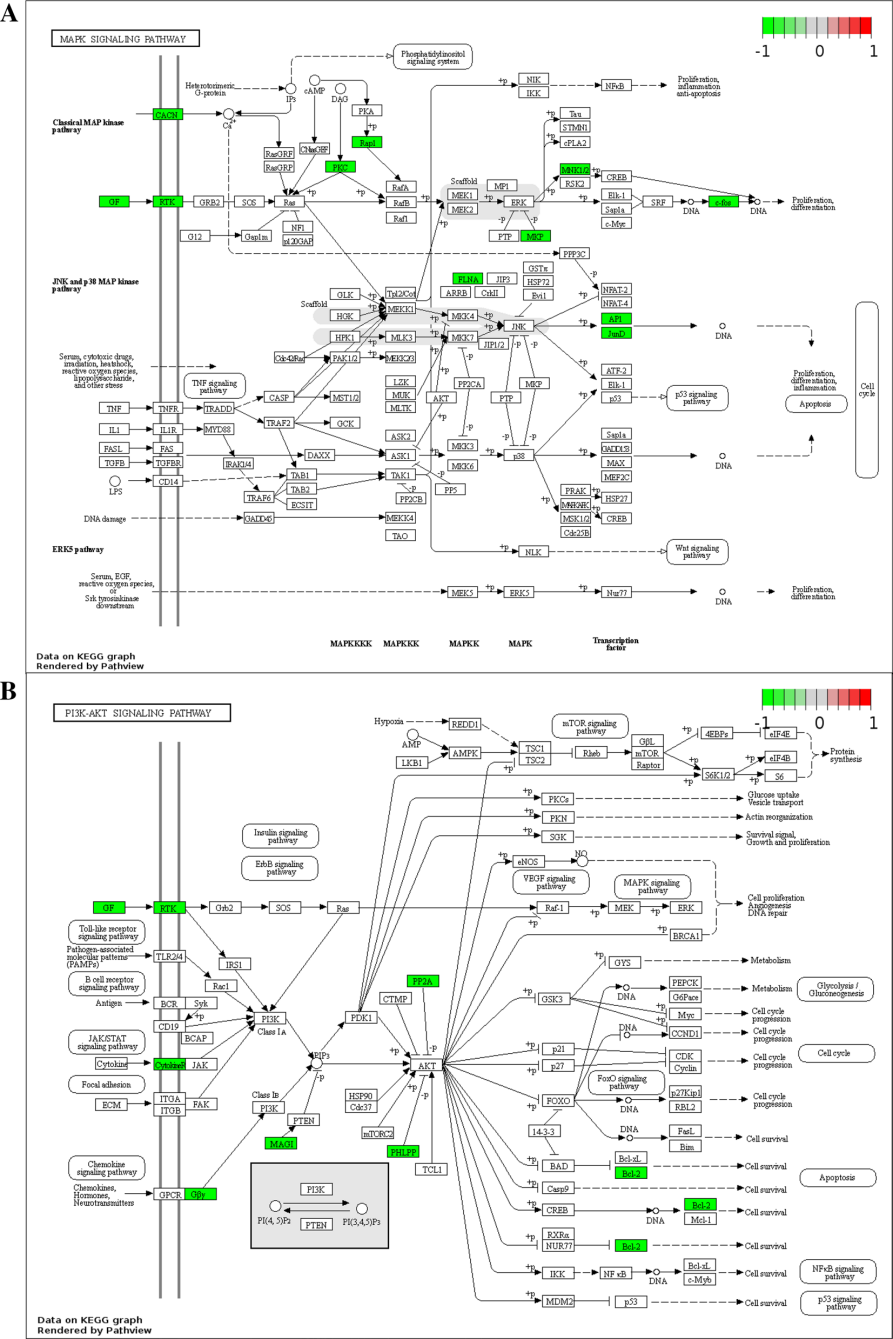
Pathway analysis and visualization of 320 target genes. The top two enriched pathways: **A** MAPK signaling pathway; **B** PI3K-AKT signaling pathway

Next, KEGG pathway enrichment analysis were performed using pathview [23] to illustrate the functional annotations of the 320 target genes. As displayed in Fig. 6, the top two enriched pathways were “MAPK signaling pathway” and “PI3K-AKT signaling pathway”, both of which were tumor-related pathways [27–30].

### Identification of fifteen hubgenes from the PPI network with MCODE and validation of their downregulation in GC and their regulation by circRNAs

**Fig. 7:**
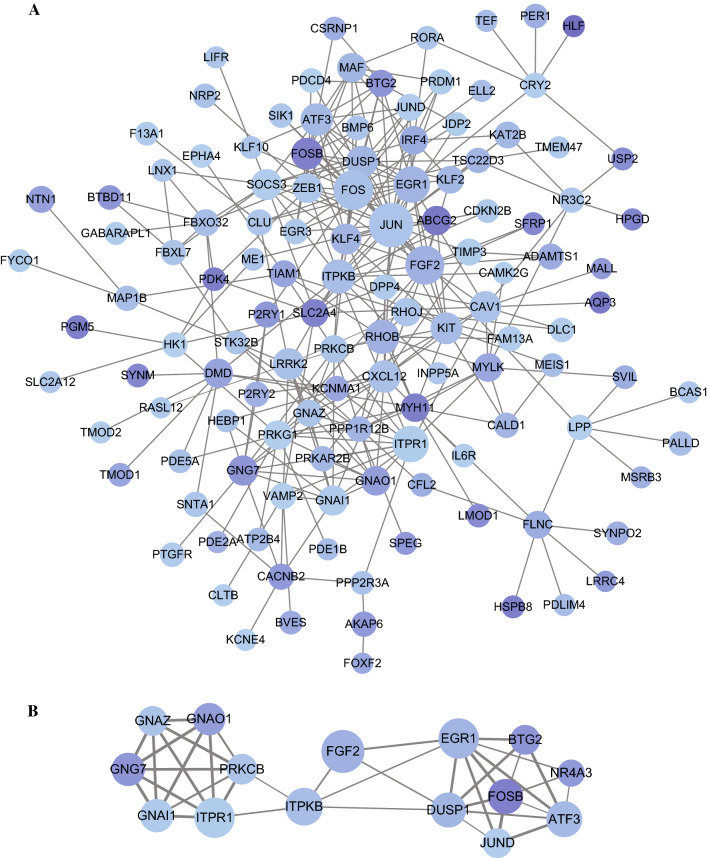
Construction of a protein–protein interaction (PPI) network with 320 target mRNAs and identification of hubgenes from the PPI network. **A** The PPI network with 320 target genes. **B** The subnetwork with 15 hubgenes that extracted from the PPI network with the “Molecular Complex Detection (MCODE)” algorithm. **A**, **B** The node color changes gradually from light blue to dark blue in ascending order according to the fold change of target genes; the thickness of line between nodes indicate the interacting proteins varies based on the combined score for the protein association

Then, a PPI network was built to analyze protein interactions with the 320 target genes. After removing unconnected nodes and nodes with connecting frequency smaller than 5, 125 nodes and 305 edges were mapped in the PPI network (Fig. 7A). Considering the key role of hubgenes in biological process, MCODE, a plugin of Cytoscape, was utilized to screen hubgenes from the PPI network. Using the criteria of that the k-core is 2, nine subnetworks were identified, and one subnetwork with highest MCODE score 5.571 that contains 15 nodes and 39 edges was selected (Fig. 7B). There were fifteen genes (ATF3, BTG2, DUSP1, EGR1, FGF2, FOSB, GNAI1, GNAO1, GNAZ, GNG7, ITPKB, ITPR1, JUND, NR4A3, and PRKCB) in this subnetwork, and these fifteen genes were identified as hubgenes.

**Fig. 8:**
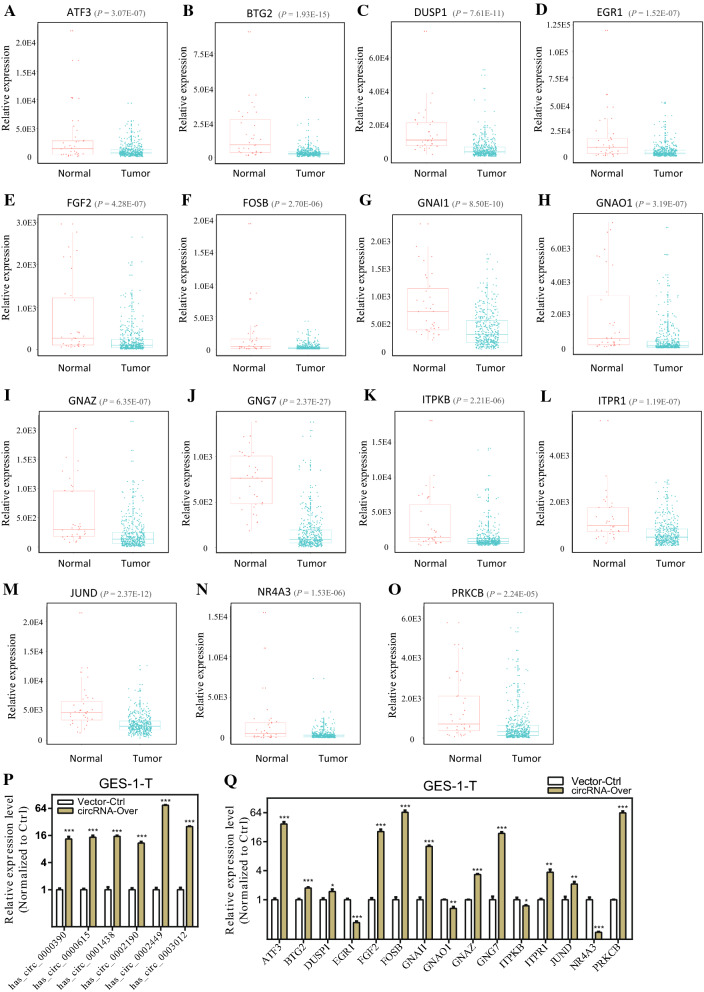
Box-scatter plots for the 15 hubgenes expression in GC from TCGA and the effect of circRNAs overexpressions on hubgenes expressions. **A**–**O** Box scatter plots for the expression of 15 hubgenes in GC from TCGA: **A** ATF3, **B** BTG2, **C** DUSP1, **D** EGR1, **E** FGF2, **F** FOSB, **G** GNAI1, **H** GNAO1, **I** GNAZ, **J** GNG7, **K** ITPKB, **L** ITPR1, **M** JUND, **N** NR4A3, and **O**PRKCB. **P**–**Q** Overexpressing plasmids of the six circRNAs were constructed and GC cells were transfected with pooled circRNA vectors. **P** The overexpression efficiency of circRNA vectors on RNA level of circRNAs were measured by qRT-PCR analysis. **Q** The mRNA levels of 15 hubgenes in GC cells transfected with circRNA vectors by qRT-PCR analysis

The expression levels of the fifteen genes in GC from TCGA were demonstrated in Fig. 8A–O, and results showed that all these fifteen hubgenes was downregulated in GC tissues compared with normal tissues. Then, RT-qPCR analysis was performed with the above-mentioned 8 pairs of GC samples and adjacent nontumor tissues to verify the induced expression of these hubgenes; the results showed that expression of the fifteen hubgenes were dramatically decreased in GC tissues than adjacent normal tissues (Additional file 2: Figure S4A–D).

In addition, to determine the effect of circRNAs on the expression of hubgenes with wet lab experiments, overexpressing plasmids of the six circRNAs were constructed and GC cells were transfected with pooled circRNA vectors. The respective increase of circRNA expressions induced by overexpressing plasmids was confirmed by qRT-PCR analysis (Fig. 8P); meanwhile, significant increases of almost all 15 hubgenes (except four of them) in mRNA level was observed (Fig. 8Q). Collectively, these results indicate that the hubgenes are modulated by circRNAs.

### Identification of three therapeutic drugs for GC based on CMap analysis and validation of their effect on GC cell proliferation and hubgenes expressions

**Fig. 9:**
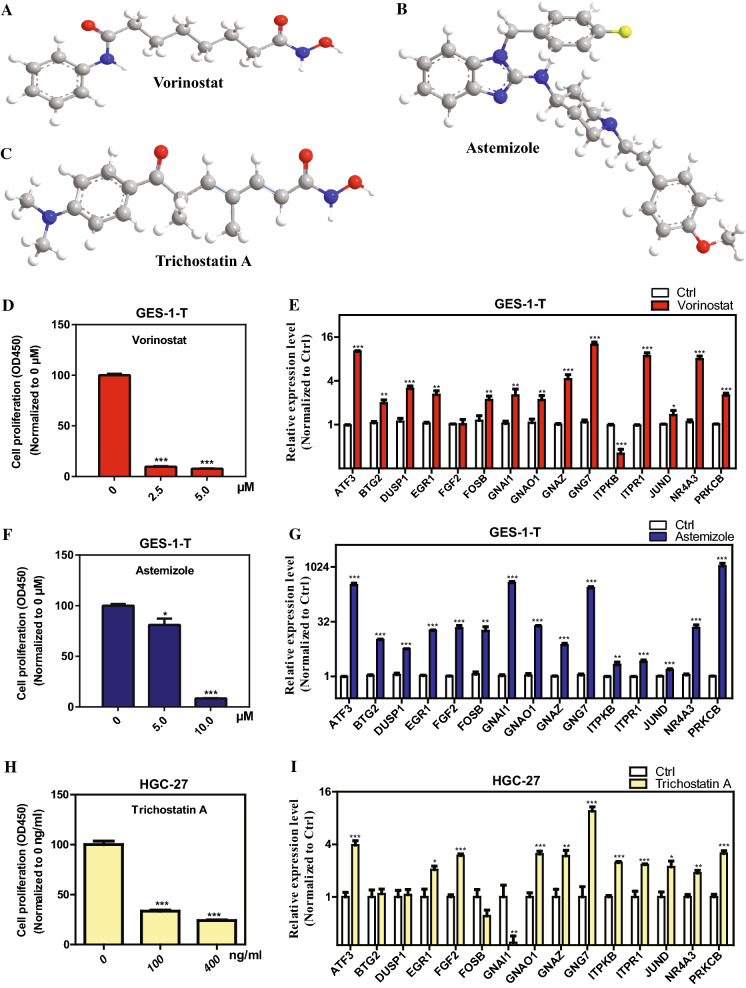
Chemical structures of the three compounds identified by the connectivity map (CMap) analysis and the effect of these three compounds on GC cell proliferation and hubgenes expressions. **A**–**C** Chemical structures of the three compounds: **A** Vorinostat, **B** Astemizole, and **C** Trichostatin A. **D**, **F**, **H** GC cells were treated with increasing concentrations of **D** Vorinostat, **F** Astemizole, and **H** Trichostatin A, and GC cell proliferations were measured by CCK-8 assays. **E**, **G**, **I** GC cells were treated with **E** Vorinostat, **G** Astemizole, and **I** Trichostatin A, and the mRNA levels of 15 hubgenes were measured by qRT-PCR analysis

CMap can be used to predict compounds that may induce or reverse a given gene expression signature, and we employed CMap to find potential compounds that could disturb the gene expression pattern of the fifteen hubgenes. Downregulated tags of the fifteen hubgenes were submitted to the CMap online tool for analysis. After the signature query, three chemicals, vorinostat, astemizole, and trichostatin A (TSA), with the most significant negative enrichment score were identified as the potential therapeutic compounds for GC (Table 4). The chemical structures of the three drugs were shown in Fig. 9A–C. The instances for each compound according to the permuted results of CMap analysis were listed in Additional file 2: Table S2; as shown, all the three compounds with different doses or in different cell lines consistently exhibited negative correlations to the query signature.

**Table 4:** Three compounds identified as treatment options for GC by CMap analysis

CMap name	Mean	n	Enrichment	*p*	Specificity	Percent non-null
Vorinostat	− 0.634	12	− 0.727	0	0.0708	100
Astemizole	− 0.621	5	− 0.766	0.00134	0.0495	100
Trichostatin A	− 0.443	182	− 0.404	0	0.3564	71

In addition, we validated whether the three bioactive drugs can alleviate GC progression and can modulate expression of hubgenes. Our results showed that treatment of GC cells with increasing concentrations of all the three drugs markedly reduced GC cell proliferation by CCK-8 assays in a dose-dependent manner (Fig. 9D, F and H), and these drugs treatments further upregulated the mRNA levels of almost all 15 hubgenes by qRT-PCR analysis (Fig. 9E, G and I). These results indicate that the three drugs predicted by CMap analysis could be used as therapeutic agents for GC.

## Discussion

CircRNAs that were first discovered in eukaryotic cells in 1979 [31] has been recently re-recognized and elicited attention. Unlike the formation of traditional linear RNA, circRNAs display a unique covalently closed circular form, which is responsible for their high stability and resistance to exonucleases. In addition, previous studies have proved their sequence conservation, abundant presence in exosomes and plasma, and spatial and cell-type specificity [32]. Thereby, circRNAs have great potential to serve as a biomarker for early diagnosis and prognosis of many human diseases including cancers. With the development of high-throughput sequencing and bioinformatics analysis, mounting circRNAs were found and confirmed to be involved in the regulation of diversified biological processes in different cancer types [33, 34]. In GC, some circRNAs, such as circHECTD1 [35] and circFAT1 [36], have been reported to be involved in GC cell proliferation, migration and invasion, and thereby exert their tumor-promoting or tumor-suppressing effects. However, the identification and function of circRNAs in GC still require further exploration.

In this study, we collected microarray and RNA-Seq datasets of GC from GEO databases and PubMed publications and screened six downregulated circRNAs (hsa_circ_0000390, hsa_circ_0000615, hsa_circ_0001438, hsa_circ_0002190, hsa_circ_0002449 and hsa_circ_0003120). Following sanger sequencing, RNase R treatment and RT-qPCR analysis, the six DECs were validated and their decreased expression in GC tissues was confirmed. Previous studies have revealed that hsa_circ_0000615 acts as miRNA sponge to exert different roles in different solid tumors [37–40]; for example, hsa_circ_0000615 upregulates Sp1 expression by adsorbing miR-150-5p, and thereby promoting the proliferation and metastasis of nasopharyngeal cancer cells [38]. However, until now studies about the effect of hsa_circ_0000615 and the other five DECs on GC have been rarely reported. Further studies are imperative to determine their potential roles in GC.

As ceRNA, circRNA harboring MREs can sponge miRNAs, which suppress the miRNA activity and result in alteration of expression level of miRNA target genes [41, 42]. Subcellular localization analysis and AGO2-binding sites mining in this study indicate that the six DECs in the cytoplasm probably sponge miRNAs. To further determine whether the above six DEC act as ceRNAs in GC, their corresponding MREs were predicted with two online tools, CircInteractome and Circbank. The former web tool forecasts MREs via Targetscan algorithm, which predicts MREs by surveying for 7-mer or 8-mer complementarity to seed region and the 3′ end of each miRNA [16, 43]. The latter web tool predicts MREs based on two different algorithms including miRanda [44] and Targetscan [43]. We selected miRNAs predicted by both CircInteractome and Circbank as target miRNAs of the six DECs, and collectively, 36 circRNA-miRNA interactions composed of 6 circRNAs and 33 miRNAs were identified.

After intersecting the target genes of the aforementioned 33 miRNAs and DEGs in GC from TCGA, 320 overlapped target genes were acquired to establish circRNA-miRNA-mRNA regulatory networks, providing an evidence of the ceRNA functional mechanism of the six DECs in GC. The KEGG pathway analysis indicated that these target genes were related to two critical tumor-associated signaling pathways, “MAPK signaling pathway” [27, 28] and “PI3K-AKT signaling pathway” [29, 30]. To comprehensively reveal potential relationships of target genes, we developed a PPI network using the 320 target genes and extracted 15 hubgenes (ATF3, BTG2, DUSP1, EGR1, FGF2, FOSB, GNAI1, GNAO1, GNAZ, GNG7, ITPKB, ITPR1, JUNID, NR4A3, PRKCB); and the differential expressions of the 15 hubgenes were subsequently validated with GC tissues from TCGA and with our GC samples. As previously demonstrated, some of the fifteen genes serve crucial roles in GC [45–47]. For example, Tang et al. have demonstrated that FOSB was significantly decreased in GC tissues, consistent with the results of this study, and moreover, downregulated expression of FOSB was correlated with poor prognosis for GC patients [46].

With the development of novel agents in recent years, survival outcomes of GC patients have improved [48]. However, the overall prognosis of patients with advanced gastric cancer remains poor, and more effective drugs against GC are needed. Therefore, CMap analysis of the fifteen hubgenes was performed to explore available compounds for the treatment of GC. Based on the genome-wide expression profiling of gene transcripts technology, CMap presents a data-driven and reliable approach for identifying new drugs or repositioning existing drugs [49]. Three chemicals (vorinostat, TSA, and astemizole) were determined and validated as the therapeutic options for GC. As histone deacetylase (HDAC) inhibitor, vorinostat alters the level of histone and nonhistone protein acetylation and thereby regulates gene expression, cell proliferation, angiogenesis and cell survival [50]. Vorinostat is FDA-approved for the treatment of cutaneous T cell lymphoma [51] and has been investigated in diverse clinical trials as a potential mono- or combination-drug therapy for solid tumors including GC [52, 53]. TSA is another kind of typical HDAC inhibitor used mainly in laboratory experiments. TSA is known to induce cell cycle arrest and apoptosis in different cancer cell lines, and its antitumor effect in solid tumors including GC has been illuminated previously [54]. Astemizole is an old anti-histamine that can target important proteins involved in the cancer progression, namely, ether à-go-go (Eag1) and Eag-related gene potassium channels [55], thus inhibiting tumor cell proliferation [56]. Moreover, previous evidence has revealed that Eag1 is expressed in several human tumor cell lines, including those from GC [57]. However, its anti-GC effect have not been elucidated presently. In this study, we found that it potentially serves as therapeutic agent for GC.

This study provides a basis for exploring the pathogenesis and treatment strategy of GC from the circRNA-miRNA-mRNA network perspective and provides more evidence for three bioactive compounds (vorinostat, TSA, astemizole) as anti-GC agents. A recent study has presented perspectives on circRNA‐miRNA‐mRNA networks to explore the pathogenesis and therapy for pancreatic ductal adenocarcinoma (PDAC) [58], and another study reported similar insights into the pathogenesis and therapy of HCC from the circRNA–miRNA–mRNA network view [59]. However, these perspectives or insights needs further wet lab investigations. Nevertheless, combined with our findings, these studies further our understanding of tumors from the perspective of circRNA‐related ceRNA networks.

## Conclusions

In conclusion, through an integrated analysis of microarray and RNA-Seq data, RT-qPCR and computational biology, six circRNA-miRNA-mRNA regulatory networks were established and these ceRNA networks uncovered six circRNAs (hsa_circ_0000390, hsa_circ_0000615, hsa_circ_0001438, hsa_circ_0002190, hsa_circ_0002449 and hsa_circ_0003120) that might function as ceRNA to play important roles in GC. Additionally, three bioactive compounds (vorinostat, TSA, astemizole) obtained from the CMap analysis were identified as therapeutic agents for GC. Our study provides a new insight for further exploration of the pathogenesis and therapy strategies of GC from the circRNA-miRNA-mRNA network perspective.

## Supplementary Information

**Additional file 1:** 1492 DEGs in GC obtained from TCGA.

**Additional file 2: Table S1.** CircRNA–miRNA interactions identified by both CircInteractome and Circbankdatabases. **Table S2**. Permuted results of the three compounds by CMap analysis. **Figure S1**. Flow chart of thepresent study. **Figure S2**. The circRNA–miRNA–mRNA regulatory networks of hsa_circ_0000615,hsa_circ_0001438, hsa_circ_0002190 and hsa_circ_0002449 in GC. **Figure S3**. The circRNA–miRNA–mRNAregulatory networks of hsa_circ_0000390 and hsa_circ_0003012 in GC. **Figure S4**. Expression of fifiteenhubgenes in GC by RT-qPCR.

## Data Availability

All data generated or analyzed during this study are included in this article. The raw data supporting the conclusions of this manuscript will be made available by the authors, without undue reservation, to any qualified researcher.

## Abbreviations

CircRNA: Circular RNA
GC: Gastric cancer
GEO: Gene Expression Omnibus
RNA-seq: RNA sequencing
DECs: Differentially expressed circRNAs
RT-qPCR: Reverse transcription-quantitative polymerase chain reaction
DEGs: Differently expressed genes
TCGA: The Cancer Genome Atlas
KEGG: Kyoto Encyclopedia of Genes and Genomes
ceRNA: Competing endogenous RNA
MRE: MiRNA response element
PPI: Protein–protein interaction
CMap: Connectivity map
CLIP: Cross-linking immunoprecipitation
MCODE: Molecular Complex Detection
HDAC: Histone deacetylase
FDA: Food and Drug Administration
