# Supplementary material for: Identification of circRNA–miRNA–mRNA networks contributes to explore underlying pathogenesis and therapy strategy of gastric cancer
Source: J Transl Med. 2021 May 28;19:226. doi: 10.1186/s12967-021-02903-5 (PMC8161999; doi:10.1186/s12967-021-02903-5)
Supplement: Supplementary file 2 — Additional file 2: Table S1. CircRNA–miRNA interactions identified by both CircInteractome and Circbankdatabases. Table S2. Permuted results of the three compounds by CMap analysis. Figure S1. Flow chart of thepresent study. Figure S2. The circRNA–miRNA–mRNA regulatory networks of hsa_circ_0000615,hsa_circ_0001438, hsa_circ_0002190 and hsa_circ_0002449 in GC. Figure S3. The circRNA–miRNA–mRNAregulatory networks of hsa_circ_0000390 and hsa_circ_0003012 in GC. Figure S4. Expression of fifiteenhubgenes in GC by RT-qPCR. [file 12967_2021_2903_MOESM2_ESM.docx]

**Supplementary Information**

**Supplementary Table 1** CircRNA–miRNA interactions identified by both CircInteractome and Circbank databases.

**Supplementary Table 2** Permuted results of the three compounds by CMap analysis.

**Supplementary Figure 1** Flow chart of the present study. GC, gastric cancer; RNA-seq, RNA sequencing; DECs, differently expressed circRNAs; AGO2, Argonaute2; MRE, miRNA response element; DEGs, differently expressed genes; TCGA, The Cancer Genome Atlas; KEGG, Kyoto Encyclopedia of Genes and Genomes; PPI, protein–protein interaction; ceRNA competing endogenous RNA; MCODE, Molecular Complex Detection; CMap, connectivity map.

**Supplementary Figure 2** The circRNA–miRNA–mRNA regulatory networks of hsa_circ_0000615, hsa_circ_0001438, hsa_circ_0002190 and hsa_circ_0002449 in GC. CircRNAs, miRNAs, and mRNAs are respectively represented by red ellipses, diamonds, and blue ellipses. (A) hsa_circ_0000615, (B) hsa_circ_0001438, (C) hsa_circ_0002190, and (D) hsa_circ_0002449.

**Supplementary Figure 3** The circRNA–miRNA–mRNA regulatory networks of hsa_circ_0000390 and hsa_circ_0003012 in GC. CircRNAs, miRNAs, and mRNAs are respectively represented by red ellipses, yellow diamonds, and blue ellipses. (A) hsa_circ_0000390. (B) hsa_circ_0003012.

**Supplementary Figure 4** Expression of fifiteen hubgenes in GC by RT-qPCR. (A) ATF3, BTG2, DUSP1, EGR1, (B) FGF2, FOSB, GNAI1, GNAO1, (C) GNAZ, GNG7, ITPKB, ITPR1, (D) JUND, NR4A3, PRKCB. GC, gastric cancer; ANT, adjacent normal tissues.

**Supplementary Table 1 CircRNA–miRNA interactions identified by both CircInteractome and Circbank databases**

| **CircRNA-miRNA interactions** | **CircInteractome** | | | | | **Circbank** | | |
| --- | --- | --- | --- | --- | --- | --- | --- | --- |
|  | **miRNA ID** | **Site Type** | **Start** | **End** | **Sites** | **miRNA ID** | **miRanda binding site** | **Targetscan  binding site** |
|  |  |  |  |  |  |  |  |  |
| hsa_circ_0000390 | hsa-miR-1248 | 8mer-1a | 281 | 288 | 1 | hsa-miR-1248 | 261 | 281 288 |
|  | hsa-miR-136 | 8mer-1a | 195 | 202 | 1 | hsa-miR-136-5p | 180 | 195 202 |
|  | hsa-miR-518a-5p | 7mer-m8 | 26 | 32 | 1 | hsa-miR-518a-5p | 14 | 26 32 |
|  | hsa-miR-527 | 7mer-m8 | 26 | 32 | 1 | hsa-miR-527 | 14 | 26 32 |
|  | hsa-miR-578 | 7mer-m8 | 324 | 330 | 1 | hsa-miR-578 | 311 | 324 330 |
|  | hsa-miR-629 | 7mer-m8 | 257 | 263 | 1 | hsa-miR-629-3p | 223 | 198 237 203 243 |
|  | hsa-miR-646 | 7mer-m8 | 303 | 309 | 1 | hsa-miR-646 | 293 | 303 309 |
| hsa_circ_0000615 | hsa-miR-1200 | 7mer-m8 | 499 | 505 | 1 | hsa-miR-1200 | 485 | 499 505 |
|  | hsa-miR-1224-3p | 8mer-1a 7mer-m8 | 107 294 | 114 300 | 2 | hsa-miR-1224-3p | 281 | 107 294 114 300 |
|  | hsa-miR-1236 | 7mer-m8 | 88 | 94 | 1 | hsa-miR-1236-3p | 76 | 88 94 |
|  | hsa-miR-1248 | 7mer-1a 8mer-1a | 603 629 | 609 636 | 2 | hsa-miR-1248 | 611 | 558 603 629 563 609 636 |
|  | hsa-miR-1272 | 7mer-m8 | 228 | 234 | 1 | hsa-miR-1272 | 211 | 228 234 |
|  | hsa-miR-142-3p | 7mer-m8 | 326 | 332 | 1 | hsa-miR-142-3p | 312 | 326 332 |
|  | hsa-miR-145 | 8mer-1a | 270 | 277 | 1 | hsa-miR-145-5p | 257 | 270 277 |
|  | hsa-miR-149 | 8mer-1a | 818 | 825 | 1 | hsa-miR-149-5p | 801 | 687 818 692 825 |
|  | hsa-miR-432 | 7mer-m8 | 456 | 462 | 1 | hsa-miR-432-5p | 444 | 456 462 |
|  | hsa-miR-487a | 7mer-m8 | 718 | 724 | 1 | hsa-miR-487a-3p | 705 | 718 724 |
|  | hsa-miR-623 | 8mer-1a | 371 | 378 | 1 | hsa-miR-623 | 357 | 371 378 |
|  | hsa-miR-767-5p | 7mer-m8 | 507 | 513 | 1 | hsa-miR-767-5p | 495 | 507 513 |
|  | hsa-miR-942 | 7mer-m8 7mer-m8 | 556 636 | 562 642 | 2 | hsa-miR-942-5p | 587 621 539 | 556 602 636 562 607 642 |

**Supplementary Table 1 CircRNA–miRNA interactions identified by both CircInteractome and Circbank databases (Continued)**

| **CircRNA-miRNA interactions** | **CircInteractome** | | | | | **Circbank** | | |
| --- | --- | --- | --- | --- | --- | --- | --- | --- |
|  | **miRNA ID** | **Site Type** | **Start** | **End** | **Sites** | **miRNA ID** | **miRanda binding site** | **Targetscan  binding site** |
|  |  |  |  |  |  |  |  |  |
| hsa_circ_0001438 | hsa-miR-942 | 7mer-m8 7mer-m8 | 176 190 | 182 196 | 2 | hsa-miR-942-5p | 177 162 | 176 190 182 196 |
| hsa_circ_0002190 | hsa-miR-323-3p | 7mer-m8 | 85 | 91 | 1 | hsa-miR-323a-3p | 71 | 85 91 |
|  | hsa-miR-513a-5p | 7mer-m8 | 283 | 289 | 1 | hsa-miR-513a-5p | 271 | 283 289 |
|  | hsa-miR-515-5p | 7mer-m8 | 52 | 58 | 1 | hsa-miR-515-5p | 35 | 52 58 |
|  | hsa-miR-598 | 7mer-m8 | 70 | 76 | 1 | hsa-miR-598-3p | 56 | 70 76 |
| hsa_circ_0002449 | hsa-miR-1200 | 7mer-m8 | 184 | 190 | 1 | hsa-miR-1200 | 166 | 184 190 |
|  | hsa-miR-1261 | 7mer-m8 | 80 | 86 | 1 | hsa-miR-1261 | 69 | 45 80 50 86 |
|  | hsa-miR-659 | 7mer-m8 | 23 | 29 | 1 | hsa-miR-659-3p | 9 | 23 91 29 96 |
| hsa_circ_0003012 | hsa-miR-1184 | 7mer-m8 | 478 | 484 | 1 | hsa-miR-1184 | 466 | 379 478 384 484 |
|  | hsa-miR-1282 | 7mer-m8 | 348 | 354 | 1 | hsa-miR-1282 | 336 | 348 354 |
|  | hsa-miR-1322 | 7mer-m8 7mer-m8 | 468 637 | 474 643 | 2 | hsa-miR-1322 | 625 456 | 468 637 474 643 |
|  | hsa-miR-140-3p | 7mer-m8 | 113 | 119 | 1 | hsa-miR-140-3p | 99 | 113 119 |
|  | hsa-miR-621 | 7mer-m8 | 670 | 676 | 1 | hsa-miR-621 | 656 | 670 676 |
|  | hsa-miR-548p | 8mer-1a | 667 | 674 | 1 | hsa-miR-548p | 653 | 667 674 |
|  | hsa-miR-548g | 7mer-m8 7mer-m8 | 427 687 | 433 693 | 2 | hsa-miR-548g-3p | 413 | 427 687 433 693 |
|  | hsa-miR-653 | 7mer-1a | 66 | 72 | 1 | hsa-miR-653-3p | 292 | 306 312 |

**Supplementary Table 2 Permuted results of the three compounds by CMap analysis**

| **Rank** | **Batch** | **cMap name** | **Dose** | **Cell** | **Score** | **Up** | **Down** | **Instance_id** |
| --- | --- | --- | --- | --- | --- | --- | --- | --- |
| 6018 | 603 | vorinostat | 10 µM | PC3 | -0.791 | -0.764 | 0.272 | 1220 |
| 6012 | 513 | vorinostat | 10 µM | MCF7 | -0.789 | -0.625 | 0.408 | 1058 |
| 5964 | 506 | vorinostat | 10 µM | MCF7 | -0.756 | -0.647 | 0.343 | 1000 |
| 5891 | 725 | vorinostat | 10 µM | MCF7 | -0.728 | -0.533 | 0.42 | 5217 |
| 5792 | 767 | vorinostat | 10 µM | MCF7 | -0.691 | -0.665 | 0.24 | 6939 |
| 5707 | 757 | vorinostat | 10 µM | MCF7 | -0.665 | -0.407 | 0.464 | 5580 |
| 5540 | 626 | vorinostat | 10 µM | MCF7 | -0.628 | -0.392 | 0.431 | 1645 |
| 5379 | 750 | vorinostat | 10 µM | HL60 | -0.595 | -0.488 | 0.291 | 6179 |
| 5017 | 727 | vorinostat | 10 µM | PC3 | -0.529 | -0.383 | 0.31 | 4444 |
| 4988 | 765 | vorinostat | 10 µM | MCF7 | -0.525 | -0.33 | 0.358 | 6980 |
| 4630 | 602 | vorinostat | 10 µM | HL60 | -0.47 | -0.36 | 0.256 | 1161 |
| 4427 | 650 | vorinostat | 10 µM | HL60 | -0.442 | -0.3 | 0.28 | 2680 |
| 6098 | 725 | trichostatin A | 100 nM | MCF7 | -0.957 | -0.789 | 0.465 | 5209 |
| 6096 | 632 | trichostatin A | 100 nM | MCF7 | -0.944 | -0.841 | 0.396 | 1471 |
| 6091 | 663 | trichostatin A | 100 nM | MCF7 | -0.908 | -0.771 | 0.418 | 2794 |
| 6085 | 647 | trichostatin A | 100 nM | MCF7 | -0.886 | -0.777 | 0.384 | 3227 |
| 6082 | 726 | trichostatin A | 100 nM | MCF7 | -0.868 | -0.618 | 0.519 | 5260 |
| 6074 | 656 | trichostatin A | 100 nM | MCF7 | -0.845 | -0.697 | 0.409 | 2835 |
| 6071 | 706 | trichostatin A | 100 nM | MCF7 | -0.838 | -0.713 | 0.384 | 4954 |
| 6063 | 637 | trichostatin A | 100 nM | MCF7 | -0.83 | -0.744 | 0.344 | 2268 |
| 6058 | 764 | trichostatin A | 100 nM | PC3 | -0.828 | -0.788 | 0.297 | 7136 |
| 6055 | 766 | trichostatin A | 100 nM | MCF7 | -0.827 | -0.648 | 0.435 | 7005 |
| 6051 | 752 | trichostatin A | 100 nM | MCF7 | -0.817 | -0.662 | 0.408 | 6085 |
| 6050 | 636 | trichostatin A | 100 nM | MCF7 | -0.816 | -0.64 | 0.429 | 2247 |
| 6041 | 704 | trichostatin A | 100 nM | PC3 | -0.808 | -0.74 | 0.319 | 4565 |
| 6031 | 506 | trichostatin A | 100 nM | MCF7 | -0.8 | -0.598 | 0.449 | 992 |
| 6027 | 705 | trichostatin A | 100 nM | MCF7 | -0.797 | -0.668 | 0.375 | 4388 |
| 6017 | 1079 | trichostatin A | 1 µM | PC3 | -0.791 | -0.78 | 0.256 | 7105 |
| 6014 | 506 | trichostatin A | 1 µM | MCF7 | -0.79 | -0.678 | 0.357 | 1014 |
| 6011 | 689 | trichostatin A | 100 nM | PC3 | -0.788 | -0.643 | 0.389 | 4072 |
| 6003 | 691 | trichostatin A | 100 nM | MCF7 | -0.783 | -0.626 | 0.4 | 4153 |
| 6002 | 701 | trichostatin A | 100 nM | PC3 | -0.781 | -0.744 | 0.28 | 4302 |
| 6013 | 744 | astemizole | 9 µM | MCF7 | -0.789 | -0.671 | 0.363 | 6807 |
| 5935 | 636 | astemizole | 9 µM | MCF7 | -0.745 | -0.464 | 0.512 | 2211 |
| 5250 | 616 | astemizole | 9 µM | PC3 | -0.568 | -0.421 | 0.323 | 2049 |
| 4989 | 728 | astemizole | 9 µM | PC3 | -0.525 | -0.29 | 0.398 | 4471 |
| 4669 | 614 | astemizole | 9 µM | HL60 | -0.477 | -0.419 | 0.205 | 1365 |


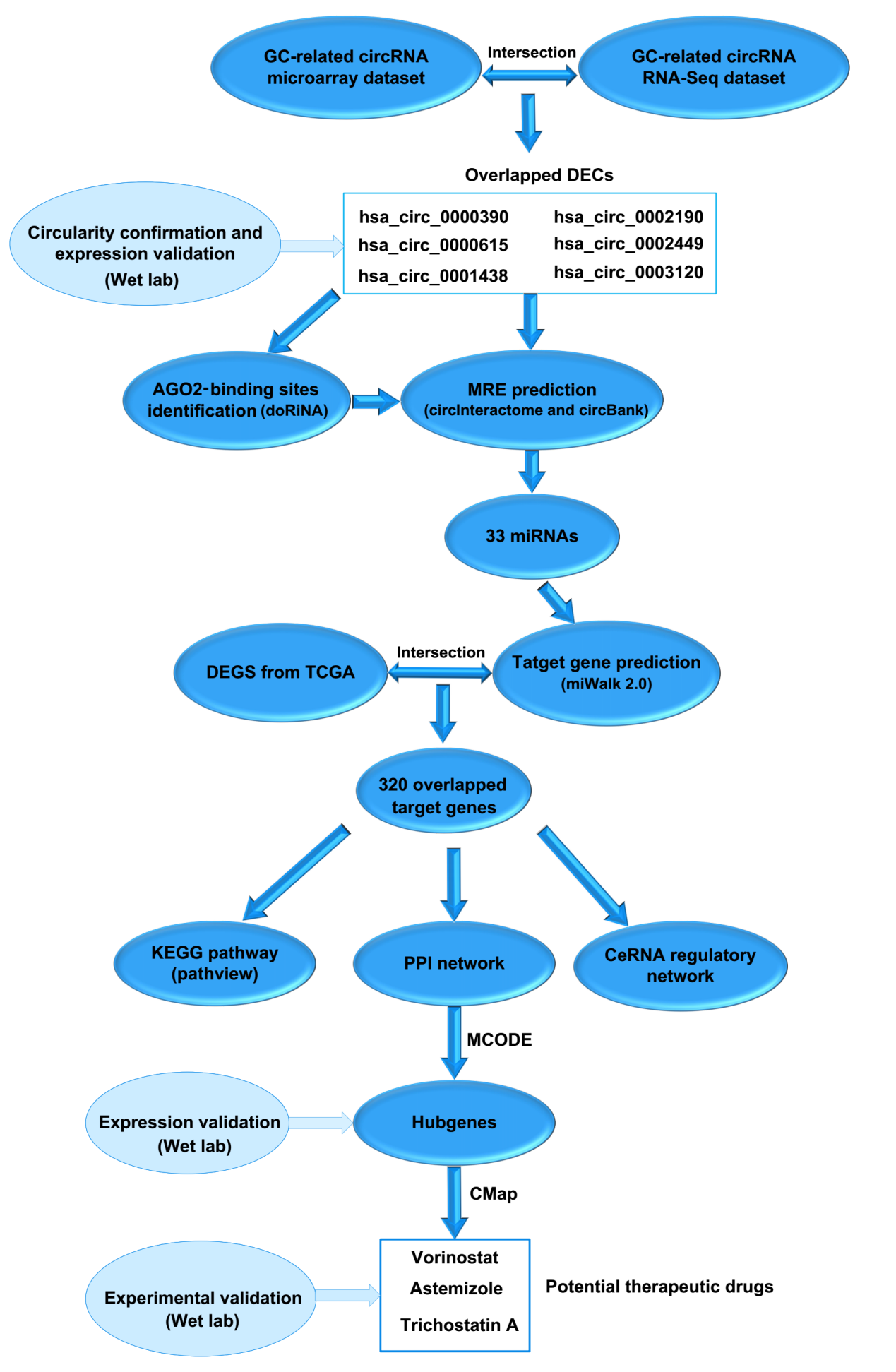


**Supplementary Figure 1**


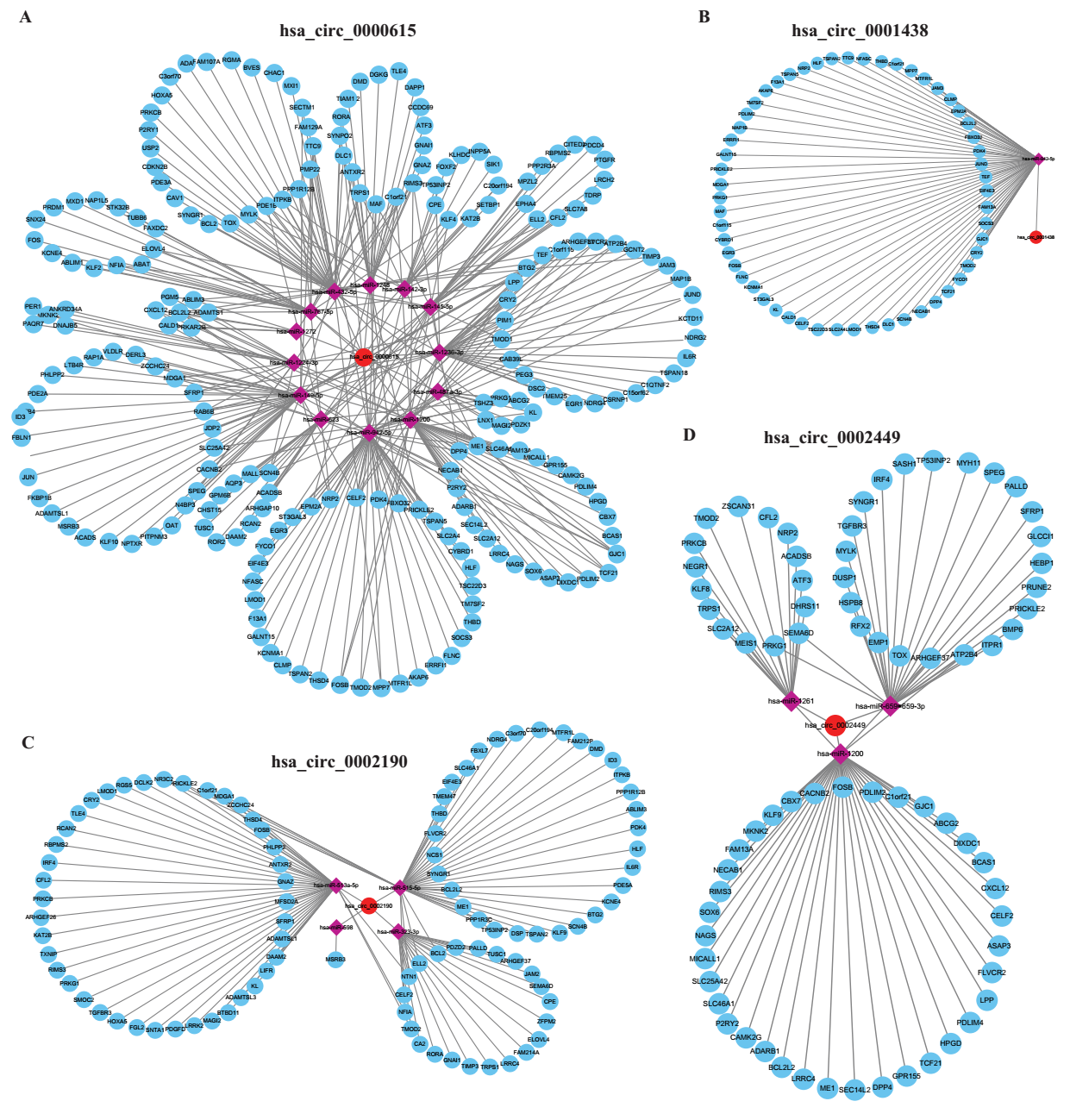


**Supplementary Figure 2**


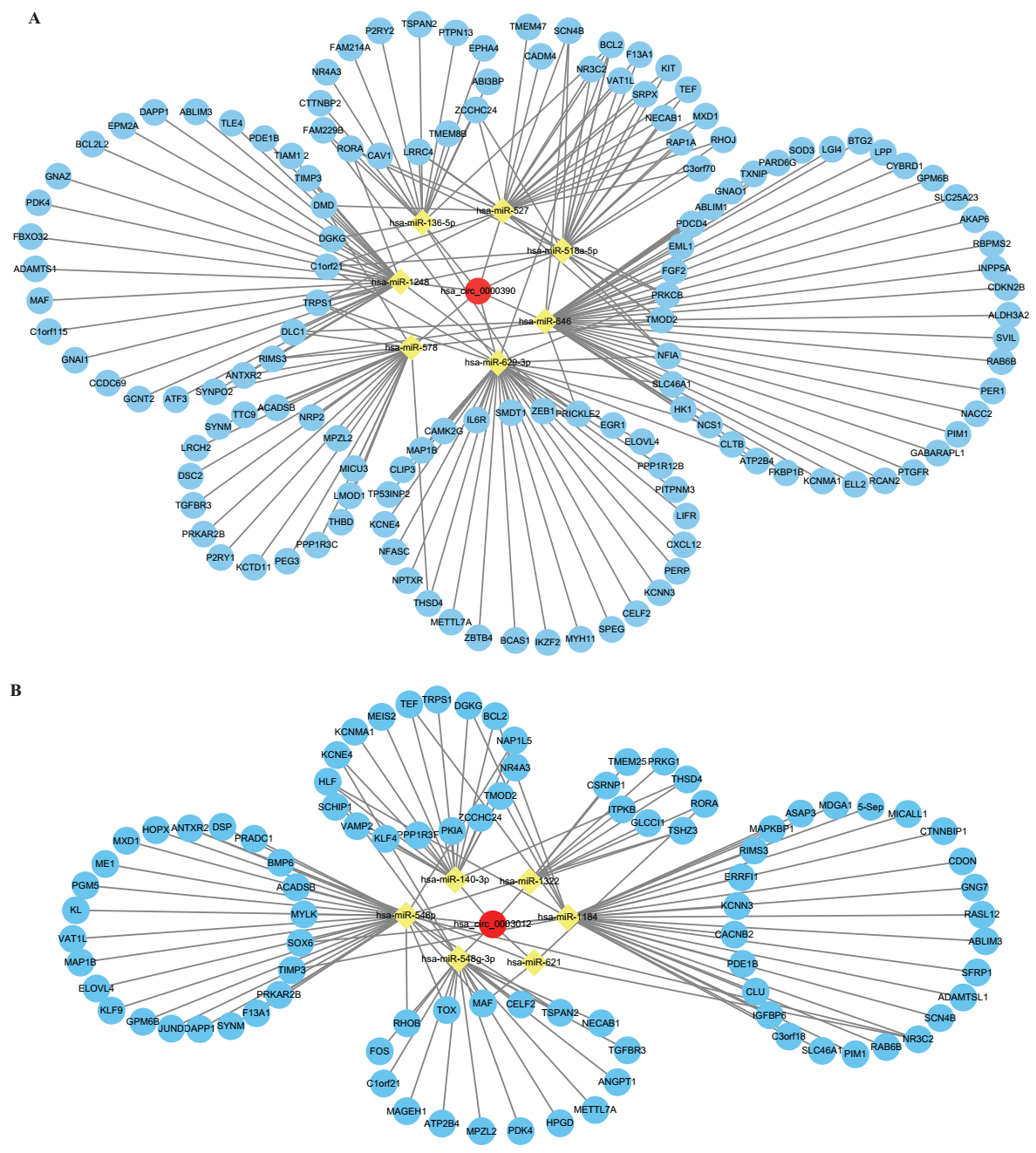


**Supplementary Figure 3**


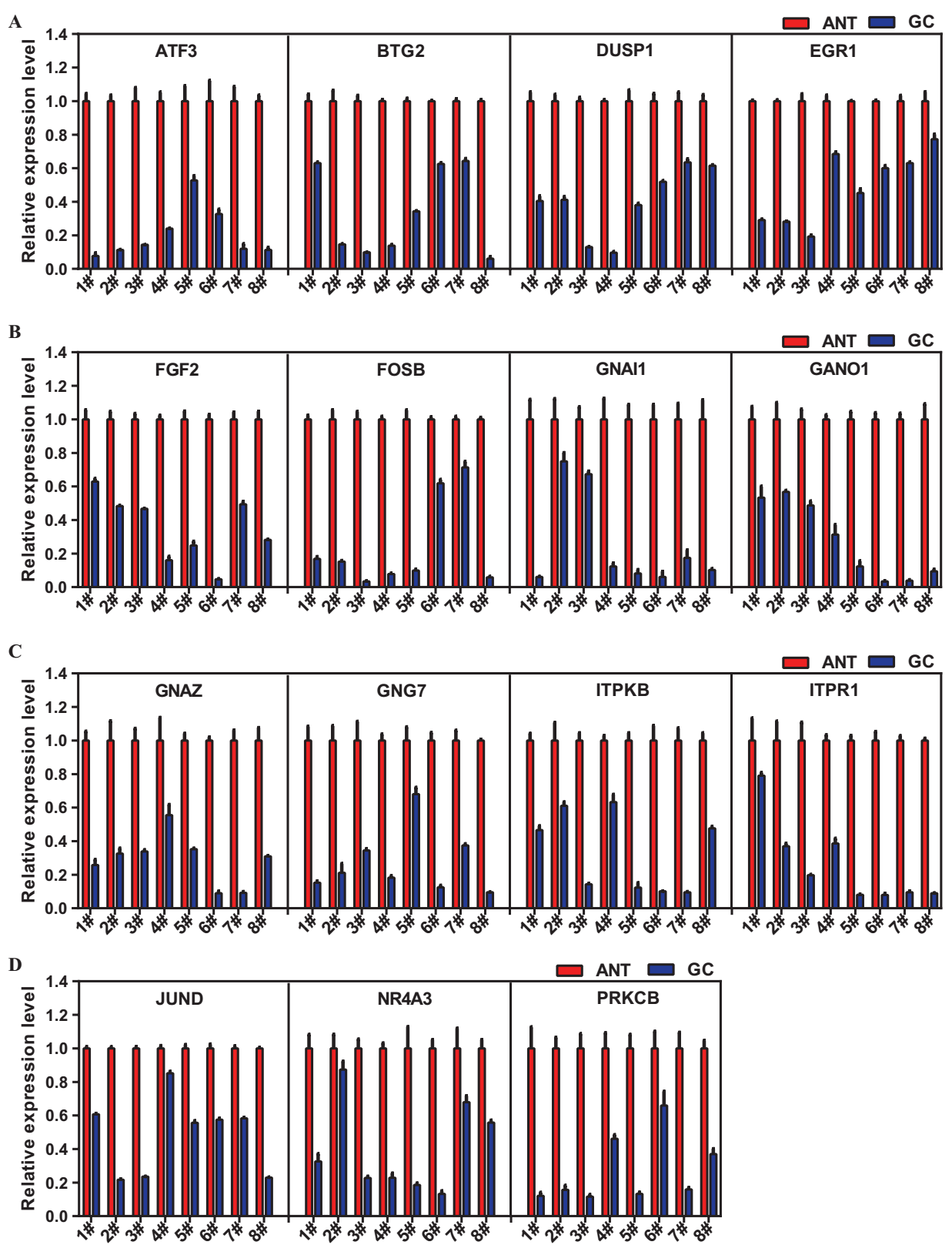


**Supplementary Figure 4**
